# Soziale Teilhabe in Pflegeheimen während der COVID-19-Pandemie („coronavirus disease 2019“): Zielvorgabe und wahrgenommene Teilhabe als Erfolgsfaktoren der Pandemiebewältigung in Altenpflegeeinrichtungen

**DOI:** 10.1007/s11553-023-01055-2

**Published:** 2023-06-12

**Authors:** Bianca Plangger, Priya-Lena Riedel, Vanessa Kulcar, Barbara Juen

**Affiliations:** 1 Institut für Psychologie, Universität Innsbruck, Universitätsstraße 5–7 (Büro), Universitätsstraße 15 (Postadresse), 6020 Innsbruck, Österreich; 2 Disaster Competence Network Austria, Wien, Österreich

**Keywords:** COVID-19, Ältere Menschen, Partizipation, Krisenkommunikation, Pflegeheime, COVID-19, Older people, Participation, Crisis communication, Nursing homes

## Abstract

**Hintergrund:**

Die COVID-19 Pandemie (“coronavirus disease 2019”) stellt für den Gesundheitsbereich eine Herausforderung dar. Zur Bewältigung dieser durch angepasste Maßnahmen ist die Einbeziehung von betroffenen Gruppen zentral.

**Ziel:**

Im Rahmen dieses Papers wird die wahrgenommene soziale Teilhabe von Mitarbeitenden in Altenpflegeeinrichtungen und Anpassungsmöglichkeiten von Maßnahmen sowie deren Bedeutung für Bewohnende und Angehörige während der Pandemie dargestellt.

**Methodik:**

Von Juni 2021 bis März 2022 wurden 26 Leitfadeninterviews und zwei Fokusgruppen mit Mitarbeitenden und Bewohnenden deutschsprachiger Pflegeheime durchgeführt.

**Ergebnisse:**

Eine klare Zielvorgabe war eine wesentliche Grundvoraussetzung der Krisenbewältigung. Teilhabeorientiertere Organisationen ermöglichten Mitarbeitenden Entscheidungsspielräume in der Zielerreichung. Hierdurch wurden mehr bedürfnisorientierte Anpassungen der Infektionsschutzmaßnahmen möglich.

**Diskussion:**

Klare Krisenstrategien durch die Führungsebene bei gegebenen Teilhabemöglichkeiten Mitarbeitender können eine erfolgreiche Krisenbewältigung in Pflegeheimen fördern. Dadurch können Maßnahmen angepasst und das Wohlbefinden aller Betroffenen geschützt werden.

## Abstract (en)

**Background:**

The COVID-19 pandemic („coronavirus disease 2019“) challenges the health sector. Involving affected groups is essential to coping with these through adaptation of measures.

**Objective:**

This study describes perceived participation of employees in care facilities for elderly and opportunities for the adaptation of measures as well as effects for residents and their relatives during the pandemic.

**Methodology:**

From June 2021 to March 2022, 26 semi structured interviews and two focus groups were conducted with staff and residents of German speaking care facilities.

**Results:**

A clear target was essential for successful crisis management. Participation-oriented organizations provided employees scopes for need-based adjustments within infection control measures within the efforts of target achievement.

**Discussion:**

Clear leadership and strategies combined with opportunities for staff to participate enhance successful crisis management in care facilities. Protective measures can be adapted and wellbeing of residents protected.

## Hintergrund

Die COVID-19-Pandemie („coronavirus disease 2019“) stellte durch ihre sich rasch verändernden Bedingungen hinsichtlich Ausbreitung, Gefährlichkeit und den entsprechenden Schutzmaßnahmen die Gesundheitssysteme vor immense Herausforderungen [2]. Es zeigte sich die Wichtigkeit, gefährdete Gruppen zu schützen [1, 11]. Dabei sind der aktive Einbezug und das Engagement relevanter Gruppen wichtig, um eine erfolgreiche Umsetzung von Schutzmaßnahmen zu erreichen [13].

In Pflegeheimen kam es während der ersten COVID-19-Welle zu einer erheblichen Isolation pflegebedürftiger Menschen [4, 16, 19], indem Gruppenaktivitäten, Besuche und andere Angebote stark eingeschränkt wurden [5]. Dies resultierte in psychischen und gesundheitlichen Problemen [7, 10, 15, 23].

Auch für Mitarbeitende stellte die Pandemie eine Belastung sowie einen moralischen Stressor dar [17]. Limitierte Personalressourcen und erhöhte Arbeitsbelastung schränkten die Betreuung ein [6, 12]. Als schützend für Bewohnende und Pflegende wurde die aktive Einbindung Mitarbeitender in die Maßnahmenanpassung wahrgenommen [9]. Dezentrale Strukturen, welche Entscheidungen an der Basis ermöglichten, und ein laufender Dialog mit Mitarbeitenden und Klient:innen waren Resilienzfaktoren [9, 18]. Eine derartige soziale Teilhabe wurde bereits vor der COVID-19-Pandemie als entscheidend für krisenrelevantes Verhalten identifiziert, indem sie Menschen dabei unterstützt, effizient auf Herausforderungen zu reagieren und die Wirksamkeitserwartung erhöht [14]. Soziale Teilhabe umfasst neben der proaktiven Einbindung in Entwicklung und Umsetzung von Maßnahmen (Partizipation), den Dialog mit Betroffenen [22]. Zudem müssen Bedürfnisse von Untergruppen sowie individuelle Hintergründe differenziert wahrgenommen und entsprechend adressiert werden (Inklusion und Adaptation). Dabei sind auch Kapazitäten und Ressourcen der Betroffenen zu berücksichtigen, und es sollte angestrebt werden, diese zu nutzen und auszubauen sowie Verantwortlichkeiten bei Planungs- und Entscheidungsprozessen zu übertragen (Nutzen lokaler Ressourcen; [22]).

Gleichzeitig war eine klare Führung während der akuten Krise unterstützend [9]. Die Pandemie erforderte eine Balance aus klaren Strategien bei gleichzeitiger Flexibilität in der Umsetzung. Dabei sind Kommunikation und Zusammenarbeit innerhalb der Organisation sowie mit Betreuten und ihren Angehörigen zentral, da sie die Zusammenarbeit fördern und eine Identifikation von Ressourcen sowie Adaptation von Maßnahmen ermöglichen [8, 12, 21]. Bei adäquaten Möglichkeiten zur Teilhabe entwickelten Pflegekräfte im Laufe der Pandemie proaktive Maßnahmen, um trotz der Herausforderungen den Bedürfnissen der Bewohnenden gerecht zu werden [12].

## Fragestellung

Die vorliegende Untersuchung befasst sich mit der Frage nach der Bedeutsamkeit von sozialer Teilhabe in Pflegeeinrichtungen während der Pandemie. Ziel ist die Erfassung der wahrgenommenen Teilhabemöglichkeiten und die Maßnahmenanpassung. Wir erwarten, dass die wahrgenommene soziale Teilhabe bei den Mitarbeitenden mit der Bedürfnisorientierung und dem Wohlbefinden der Bewohnenden und Angehörigen zusammenhängt.

## Studiendesign und Untersuchungsmethoden

Die Datenerhebung wurde im Rahmen des Projekts CAVE (Community Engagement und Vulnerabilitäten in der Bewältigung von Pandemien) durchgeführt, das eine präventive Erfassung von Vulnerabilitäten und Resilienzen sowie die Verbesserung der Einbindung vulnerabler Gruppen während Epidemien zum Ziel hat. Der Fokus unserer Teilstudie liegt auf Pflegewohnheimen. Von Juni 2021 bis März 2022 wurden hierfür Interviews und Fokusgruppen mit Pflegekräften, Bewohnenden und Angehörigen Erhebungen durchgeführt. Details zu Stichprobe und Untersuchungsdesign können dem Supplement entnommen werden. Es erfolgten 26 Leitfadeninterviews mit Pflegekräften, Bewohnenden und Angehörigen aus Österreich, Deutschland und Südtirol (Italien). Auch Führungskräfte, die selbst aktiv mit Bewohnenden arbeiteten, wurden als Pflegekräfte inkludiert. Der teilstrukturierte Leitfaden orientierte sich am subjektiven Erleben der Pandemie sowie der wahrgenommenen sozialen Teilhabe. Die Interviews wurden vor Ort im Pflegewohnheim oder online durchgeführt und dauerten zwischen 30 und 90 min. Zusätzlich wurden zwei Fokusgruppen mit jeweils 4 Pflegekräften bzw. 4 Bewohnenden durchgeführt. Die Fokusgruppen wurden vor Ort im Pflegewohnheim durchgeführt und dauerten zwischen 30 und 60 min. Insgesamt wurden Personen aus 13 Pflegeeinrichtungen befragt. Tab. 1 gibt einen Überblick über die Stichprobe.

**Tab. 1:** Stichprobenbeschreibung der Interviews und Fokusgruppen

**Interviewte**	*Funktion*	*Geschlecht*	*Alter (Jahre)*	*Gesamt (n)*
Leitung	Pflegedienstleitung	3 w, 2 m	34–54	5
	Abteilungsleitung	0 w, 1 m	65	1
	Stationsleitung	1 w, 0 m	47	1
Mitarbeitende	Diplompfleger:innen	1 w, 2 m	40–49	3
	Pflegeassistent:innen	4 w, 0 m	30–52	4
	Sozialarbeiter:innen	1 w, 0 m	41	1
Bewohnende	–	2 w, 2 m	61–87	4
Angehörige	–	4 w, 3 m	55–75	7
Gesamt				26
**Fokusgruppe**				
Mitarbeitende	Pflegeassistent:innen	3 w, 1 m	38–49	4
Bewohnende	–	3 w, 1 m	85–93	4
Gesamt				8

*w* weiblich, *m* männlich

Es liegt die Zustimmung des lokalen Review Boards für ethische Fragen der wissenschaftlichen Forschung vor. Die Studie folgt den Richtlinien der Deklaration von Helsinki 1975. Vor Beginn aller Erhebungen wurden die Teilnehmenden hinsichtlich ihrer Rechte, der Freiwilligkeit der Teilnahme, der Aufzeichnung des Gesprächs, einer Pseudoanonymisierung sowie Nutzung der Daten aufgeklärt und willigten der Teilnahme schriftlich ein.

Die Daten wurden mit der Software für qualitative Datenanalyse MAXQDA kodiert und eine qualitative Inhaltsanalyse nach Mayring [3] durchgeführt. Die Interviewauswahl wurde darauf ausgelegt, dass sich das Datenmaterial maximal kontrastiert. Im Folgenden wurden basierend auf dem axialen Kodieren der Grounded Theory [20] Schlüsselkategorien gebildet.

## Ergebnisse

Im Folgenden werden die Ergebnisse anhand der Schlüsselkategorien beschrieben.

1. *Wahrgenommene soziale Teilhabe bei klarer Zielvorgabe* beschreibt das Ausmaß klarer Ziele von Seiten der Führungsebene sowie die Ermöglichung einer sozialen Teilhabe durch die Pflegenden. Die Kategorie stellt den wahrgenommenen Entscheidungsspielraum von Pflegekräften und deren Einbindung in Planung und Maßnahmenumsetzung dar.
2. *Wahrgenommene Flexibilität und Bedürfnisorientierung *beschreibt die Umsetzung von Infektionsschutzmaßnahmen unter Anpassung an die Bedürfnisse der Bewohnenden und Angehörigen.

### Wahrgenommene soziale Teilhabe bei klarer Zielvorgabe

Ein wichtiger Aspekt in allen Einrichtungen war funktionierende Zusammenarbeit innerhalb der Führungsebenen und erfolgreiche Krisenplanung. Das zeigte sich z. B. darin, dass bereits vor der behördlichen Anordnung des Lockdowns Krisenpläne für Infektionskrankheiten bestanden. Klare Zielvorgaben waren in allen Einrichtungen bis auf eine vorhanden. In der wahrgenommenen sozialen Teilhabe zeigte sich eine größere Variation innerhalb der 13 untersuchten Einrichtungen.

In vier Einrichtungen hatten die Mitarbeitenden breite Möglichkeiten zur sozialen Teilhabe während der Pandemie. Dort kam es regelmäßig zum Austausch und Dialog aller Beteiligten im Pflegewohnheim. Der Fokus lag auf wertschätzender Kommunikation, Lebensqualität, Gemeinschaft, Entscheidungskompetenzen und Teilhabe von Mitarbeitenden, Bewohnenden und Angehörigen. Es fand eine Orientierung an humanitären Werten statt. Führungskräfte förderten ein respektvolles Miteinander, Unterstützung und Wertschätzung, um Zusammenarbeit sicherzustellen. Beispielsweise wurde einer Spaltung beim Thema Impfung proaktiv entgegengewirkt: „Und es ist auch von der Hausleitung so kommuniziert worden, dass sie es auch nicht wünscht [dass] es da Ausgeschlossenheiten im Team gibt, nur weil sich Menschen nicht impfen lassen“ (Mitarbeitende 01). Eigeninitiative und Austausch auf allen Ebenen bei der Umsetzung von Schutzmaßnahmen wurde gefördert. Entscheidungen wurden „im Team besprochen und dann gemeinsam entschieden“ (Mitarbeitende 31). Gleichwohl bestand das Ziel, möglichst keine COVID-19-Erkrankung in den Pflegewohnheimen zuzulassen und die Bewohnenden, sowie Mitarbeitenden bei größtmöglicher Freiheit bestmöglich zu schützen.

In 8 weiteren Einrichtungen zeigte sich zwar klare Zielvorgabe durch Führungskräfte, Teilhabemöglichkeiten fehlten jedoch. Vorgaben von Führungskräften wurden an Mitarbeitende weitergegeben, ohne diese einzubeziehen „Also die Entscheidungen haben wir mal definitiv nicht beeinflussen können“ (Fokusgruppe Mitarbeitende). Dialog wurde abgelehnt und Entscheidungen durch die Führung getroffen „Unser Heimleiter ist da sehr rigoros, der duldet keine Widerworte“ (Mitarbeitende 17). Kontrolle und Sanktionen schränkten die Mitarbeitenden ein, z. B. Test- und Maskenpflicht oder Kündigung von Ungeimpften. Sanktionen erfolgten auch durch eine Spaltung innerhalb der Belegschaft „… [hat] er angeordnet, dass alle während der gesamten Schulungszeit von neun Uhr früh bis um fünf Uhr abends die Maske durchweg zu tragen haben, weil sich im Raum drei ungeimpfte Personen befinden und die besonders gefährlich sind“ (Mitarbeitende 17). Die Führungskräfte nutzten eine utilitaristische Werteorientierung mit dem Ziel, Infektionen zu vermeiden und die Impfquote zu steigern. Eine Führungskraft drückt das so aus „Wie soll ich sagen, das war so eine Ambition von mir: ‚Wir schaffen das, dass dann herinnen niemand infiziert wird.‘ […] Ich könnte mir vorstellen, dass ich, dass ich das […] als persönliche Niederlage empfunden hätte“ (Leitung 19).

In einer Einrichtung fehlten klare Zielvorgaben, was zu eigenmächtigen Entscheidungen des Personals führte. Ein kollektiver Prozess des Abwägens zwischen Sicherheit und Lebensqualität der Bewohnenden konnte nicht stattfinden. Die Pandemiebewältigung bestand in der ad hoc Orientierung von Tag zu Tag. „Wirklich, es war jeden Tag, wir haben jeden Tag alles geplant für heute, nicht für Morgen. Morgen ist Morgen. Heute ist heute. Schauen wir wie wir weitergehen können“ (Leitung 46).

### Wahrgenommene Flexibilität und Bedürfnisorientierung

Die Bewohnenden waren angewiesen auf die bedürfnisorientierte Maßnahmenumsetzung durch die Pflegekräfte. Entsprechend war die Stimmung innerhalb der Pflegeheime stark abhängig von der jeweiligen Maßnahmenumsetzung und unterschied sich auch innerhalb von Heimen zwischen einzelnen Stationen „Wohl das ist hier gut, gell, im ersten Stock haben wir viel Unterhaltung.“ „Wir da unten weniger“ (Fokusgruppe Bewohnende). Entsprechend zeigte sich eine große Variabilität der Maßnahmenumsetzung in den untersuchten Heimen. Besonders teilhabeorientierte Einschränkungen waren gekennzeichnet durch Flexibilität, Kreativität und Bedürfnisorientierung in der Umsetzung von Infektionsschutzmaßnahmen.

#### Einschränkung der Bewegungsfreiheit.

In Einrichtungen mit weniger sozialer Teilhabe wurden Einschränkungen wie Isolation Bewohnender im Einzelzimmer oder dem eigenen Wohnbereich mit dem expliziten Verbot das Pflegewohnheim zu verlassen unterschiedslos umgesetzt. Bewohnende beschreiben Einschränkungen der Bewegungsfreiheit als extrem belastend, hielten sich jedoch oft aus Angst vor Konsequenzen oder wegen Abhängigkeiten aufgrund körperlicher Einschränkungen an Ausgehverbote: „Hab ich mich nicht getraut […] Und rausgekommen wären wir ja sowieso nicht […] weil ich bin ja angewiesen auf einen Transport“ (Bewohnende 04). Teilhabeorientiertere Einrichtungen passten die Maßnahmen stärker an. Statt Verboten und Zwang wurde auf Apelle gesetzt. Alternativen wie kontrollierte Spaziergänge wurden angeboten. Das „Einsperren“ wurde ethisch und rechtlich abgelehnt: „Das Haus verlassen können die Bewohner ja auch so. Wir haben ja gar nicht das Recht das zu verbieten“ (Leitung *11*).

#### Besuchsverbot.

Während der ersten Pandemiewelle wurde ein generelles Besuchsverbot in fast allen Einrichtungen ausgesprochen. Dies wurde als starke Belastung erlebt „Ich darf nicht mehr raus, es darf niemand rein und das war furchtbar“ (Bewohnende 04). Bewohnende mit starken kognitiven oder körperlichen Einschränkungen sowie deren Angehörige waren besonders belastet „Also mein Vater war Schlaganfallpatient […] fast blind, schwerhörig und das war für ihn eine Katastrophe im Nachhinein, weil er einfach völlig isoliert war“ (Angehörige 27). Im weiteren Verlauf gab es Anpassungen, die unterschiedlich schnell und weitgreifend umgesetzt wurden. Sicherheitsvorkehrungen zur Ermöglichung von Besuchen umfassten z. B. Beschränkung der Anzahl Besuchender, Temperaturkontrolle und Voranmeldung, Eskortierung und Überwachung, Tragen von Mund-Nasen-Schutz: „Wir haben jetzt, bis Ende Mai, nur in Begegnungszonen haben sie sich begegnen dürfen. Also mit totaler Registrierung und wirklich kompletter Kontrolle, ob, Test und so weiter. Inzwischen sind wir bei Selbstregistrierung und stichprobenartigen Kontrollen, ob die Gs eingehalten sind“ (Leitung 11). Auch Besucherboxen wurden genutzt (Glasscheibe und kein physischer Kontakt). Diese wurde von Bewohnenden meist negativ empfunden „Die Boxen mag ich nicht, weil erstens hast du da drin so schreien müssen, dass das ganze Haus mithorcht […] und zweitens war’s eh immer besetzt“ (Bewohnende 04). Als Alternative zu Besuchen wurde in verschiedenen Heimen auch auf technische Lösungen wie (Video‑)Telefonie zurückgegriffen. Diese Kontaktform wurde aber weder von Bewohnenden noch von deren Angehörigen als adäquater Ersatz empfunden. Teilhabeorientierte Organisationen strebten daher eine möglichst frühzeitige Öffnung für kontrollierte Besuche an, während andere Einrichtungen bis zu ein Jahr lang für Besuchende geschlossen blieben. Das Besuchsverbot wurde in restriktiven Heimen auch bei palliativen Bewohnenden strikt umgesetzt „Die sind alle alleine gestorben und alleine geblieben“ (Leitung 46), während in teilhabeorientierten Organisationen hierfür großzügige Ausnahmen gemacht wurden „Und die Bewohner, die verstorben sind, da haben wir das eigentlich sehr gut hingekriegt, schon mit großen Mühen, dass wir das wirklich doppelt und dreifach getestet, genesen und sonst was, dass die Angehörigen auch da sein haben können“ (Mitarbeitende 01).

#### Gruppenaktivitäten.

Gruppentherapien, Bewegung mit Musik oder Gedächtnistraining für Bewohnende wurden in Einrichtungen mit wenig sozialer Teilhabe stark eingeschränkt oder ausgesetzt „Das war alles weg damals“ (Mitarbeitende 34). Es gab jedoch auch Anpassungsbemühungen, beispielsweise durch die Verlagerung auf Einzelsettings bei den Bewohnenden im Zimmer „Wir haben insofern darauf reagiert, dass wir versucht haben, die Bewohner natürlich mehr in den Zimmern zu beschäftigen“ (Mitarbeitende 17). Andere ermöglichten weiterhin Gruppenaktivitäten unter Anpassungen und Vorsichtsmaßnahmen wie reduzierte Gruppengrößen, Übungen ohne physischen Kontakt und Mindestabstände oder im Freien. Eine Einrichtung berichtete davon, ohne besondere Vorkehrungen das Programm mit Therapien und Bewegung für Bewohnende anzubieten „Alles haben wir gehabt, alles. Sie haben mitgetanzt auch gehabt, alles. Das machen sie sehr gerne“ (Leitung 33). Bewohnende berichteten insbesondere von der Bedeutung von Ritualen und Festen, welche auch die Stimmung während der Krise aufhellte „Die Adventszeit, wir haben Zusammenkunft drüben im Gang und da wird gesungen und da wird auch wieder mal irgendwie gelacht“ (Fokusgruppe Bewohnende).

#### Informationsvermittlung.

In teilhabeorientierten Einrichtungen wurden pandemiespezifische Informationen an Angehörige und Bewohnende schriftlich sowie im Dialog durch Bewohnendentreffen bzw. in Angehörigengesprächen vermittelt. Nicht teilhabeorientierte Einrichtungen vermieden den Dialog. Eine Einrichtung berichtete davon, bewusst Information vorzuenthalten, um Bewohnende in die gewünschte Richtung zu lenken „Da hätte man dann jeden aufklären müssen, dass das jetzt eine Off-label-Impfung ist. Das haben wir natürlich nicht gemacht“ (Leitung 12). Viele Informationen waren den Bewohnenden durch kognitive oder physische Einschränkungen nicht zugänglich, wenn die Informationsbereitstellung nicht angepasst wurde „Können teilweise aufgrund von Augenerkrankungen nicht mehr lesen. Können aufgrund kognitiver Einschränkungen überhaupt nicht verstehen, um was es geht.“ (Leitung 23).

## Diskussion

Diese Untersuchung zeigt die Bedeutung wahrgenommener sozialer Teilhabe von Pflegeheimmitarbeitenden für die Anpassung von Pandemiemaßnahmen an die Bedürfnisse Bewohnender und Angehöriger. Während die Verallgemeinerbarkeit durch das Untersuchungsdesign eingeschränkt ist, bieten die Ergebnisse wertvolle Einblicke in die Situation in Pflegeheimen während der COVID-19-Pandemie. In Einrichtungen mit viel sozialer Teilhabe konnten zentrale Werte wie Freiheit der Bewohnenden trotz Einschränkungen weitgehend aufrechterhalten werden. Fehlten Teilhabemöglichkeiten, wurden Maßnahmen rigider umgesetzt, was mit stärkerer Einschränkung und Belastung der Bewohnenden einherging. Können Pflegende nicht auf die Bedürfnisse der Betreuten eingehen, erhöht das moralischen Stress und beeinträchtigt das Befinden [17]. Neben der Teilhabe stellt die klare Zielvorgabe in den Einrichtungen eine Ressource dar. Die Anpassung an die Krisensituation kann bestmöglich stattfinden, wenn durch die Führungsebene klare Ziele und Strategien vorgegeben werden, deren Umsetzung aber flexibel und bedürfnisorientiert stattfinden sollte. Es gab in der Stichprobe eine Einrichtung, in der keine Entwicklung von Krisenplänen und in der Folge auch keine Adaptation von Maßnahmen stattfand. Hier wurden die Bewegungsfreiheit sowie soziale Kontakte Bewohnender über einen langen Zeitraum massiv eingeschränkt, unabhängig von der persönlichen, gesundheitlichen oder pandemischen Situation. Dies hat, wie bereits mehrfach gezeigt wurde, negative Auswirkungen auf das Wohlbefinden und die Gesundheit der Bewohnenden [7, 15, 23]. Die meisten Einrichtungen bemühten sich, die Maßnahmen so anzupassen, dass die Einschränkungen und Belastungen für Bewohnende reduziert wurden. Soziale Teilhabe ermöglichte dabei umfangreichere und kreativere Lösungen. Dabei erwies sich der kontinuierliche Dialog auf allen Ebenen und Zielgruppen als bedeutsam. Indem der Fokus auf einem kollegialen Austausch auf allen Ebenen des Pflegewohnheim gelegt wurde sowie auf breite Entscheidungskompetenzen aller Beteiligten konnte ein Klima des respektvollen Miteinanders und der gegenseitigen Unterstützung und Wertschätzung erhalten werden, das Eigeninitiative und Austausch ermöglichte [13].

## Fazit für die Praxis

- Klare Zielvorgabe und ein gemeinsam erstellter Krisenplan sind notwendig zur Krisenbewältigung.
- Der Fokus auf soziale Teilhabe für Mitarbeitende ist zentral und fördert Anpassungsfähigkeit und Bedürfnisorientierung.
- Zeitnahe, konkrete Informationen sollten schnell weitergeleitet werden, um Unsicherheit zu reduzieren und Handlungsfähigkeit herzustellen.
- Der Dialog mit Mitarbeitenden, Angehörigen und Bewohnenden ermöglicht den gegenseitigen Austausch und lässt notwendige Anpassungen zu.
- Emotionales und psychosoziales Wohlbefinden Mitarbeitender und Bewohnender hängt zusammen.

## References

[1] Ainuddin S, Routray JK (2012) Community resilience framework for an earthquake prone area in Baluchistan. Int J Disaster Risk Reduct 2:25–36

[2] Anand U, Cabreros C, Mal J, Ballesteros F Jr, Sillanpää M, Tripathi V, Bontempi E (2021) Novel coronavirus disease 2019 (COVID-19) pandemic: from transmission to control with an interdisciplinary vision. Environ Res 197:111126 33831411 10.1016/j.envres.2021.111126 PMC8020611

[3] Baur N, Blasius J (2014) Handbuch Methoden der empirischen Sozialforschung. Springer

[4] Gangnus A, Hering C, Kohl R, Henson CS, Schwinger A, Steinhagen-Thiessen E, Kuhlmey A, Gellert P (2021) Covid-19-Schutzmaßnahmen und Einschränkungen des sozialen Lebens in Pflegeheimen. Pflege 35(3):133–142 10.1024/1012-5302/a000854 34894714

[5] Gangnus A, Hering C, Kohl R, Henson C‑S, Schwinger A, Steinhagen-Thiessen E, Kuhlmey A, Gellert P (2022) Social participation in nursing homes with Covid-19 protection measures in the second pandemic wave? Linkage of prescriptions and survey. Pflege 36(3):168–178 10.1024/1012-5302/a000898 35997038

[6] Hossain F, Clatty A (2021) Self-care strategies in response to nurses’ moral injury during COVID-19 pandemic. Nurs Ethics 28(1):23–32 33124492 10.1177/0969733020961825 PMC7604672

[7] Luchetti M, Terracciano A, Aschwanden D, Lee JH, Stephan Y, Sutin AR (2020) Loneliness is associated with risk of cognitive impairment in the Survey of Health, Ageing and Retirement in Europe. Int J Geriatr Psychiatry 35(7):794–801 32250480 10.1002/gps.5304 PMC7755119

[8] Maunder R, Hunter J, Vincent L, Bennett J, Peladeau N, Leszcz M, Sadavoy J, Verhaeghe LM, Steinberg R, Mazzulli T (2003) The immediate psychological and occupational impact of the 2003 SARS outbreak in a teaching hospital. Cmaj 168(10):1245–1251 12743065 PMC154178

[9] McCleskey JA (2014) Situational, transformational, and transactional leadership and leadership development. J Bus Stud Q 5(4):117

[10] Müller O, Neuhann F, Razum O (2020) Epidemiology and control measures in COVID-19. Dtsch Med Wochenschr [internet] 145(10):670–674 32344440 10.1055/a-1162-1987 PMC7295278

[11] Osborne J, Paget J, Giles-Vernick T, Kutalek R, Napier D, Baliatsas C, Dückers M (2021) Community engagement and vulnerability in infectious diseases: A systematic review and qualitative analysis of the literature. Soc Sci Med 284:114246 34311391 10.1016/j.socscimed.2021.114246

[12] Palacios-Ceña D, Fernández-Peña R, Ortega-López A, Fernández-Feito A, Bautista-Villaécija O, Rodrigo-Pedrosa O, Arnau-Sánchez J, Lizcano-Álvarez Á (2021) Long-term care facilities and nursing homes during the first wave of the COVID-19 pandemic: a scoping review of the perspectives of professionals, families and residents. IJERPH 18(19):10099 34639401 10.3390/ijerph181910099 PMC8508277

[13] Paton D (2000) Emergency Planning: Integrating community development, community resilience and hazard mitigation. J Am Soc Prof Emerg Manag 7(1):109–118

[14] Paton D, Millar M, Johnston D (2001) Community resilience to volcanic hazard consequences. Nat Hazards 24(2):157–169

[15] Plangger B, Unterrainer C, Kreh A, Gatterer G, Juen B (2022) Psychological effects of social isolation during the COVID-19 pandemic 2020. Geropsych: J Gerontopsychology Geriatr Psychiatry 35(1):17–29

[16] Räker M, Klauber J, Schwinger A (2021) Pflegerische Versorgung in der ersten Welle der COVID-19-Pandemie Pflege-Report. Springer, Berlin, Heidelberg, S 33–58

[17] Riedel P‑L, Kreh A, Kulcar V, Lieber A, Juen B (2022) A scoping review of moral stressors, moral distress and moral injury in healthcare workers during COVID-19. IJERPH 19(3):1666 35162689 10.3390/ijerph19031666 PMC8835282

[18] Riedel-Heller S, Richter D (2021) Psychological consequences of the COVID-19 pandemic in the general public. Public Health Forum 29(1):54–56

[19] Schweickert B, Klingeberg A, Haller S, Richter D, Schmidt N, Sin AM, Eckmanns T (2021) COVID-19-Ausbrüche in deutschen Alten- und Pflegeheimen. Epidemiologisches Bulletin. 10.25646/8174

[20] Strauss AL, Corbin JM, Niewiarra S, Legewie H (1996) Grounded theory: Grundlagen qualitativer sozialforschung. Beltz, Psychologie-Verlag-Union, Weinheim

[21] Teece DJ (2014) The foundations of enterprise performance: Dynamic and ordinary capabilities in an (economic) theory of firms. AMP 28(4):328–352

[22] Unicef (2020) Minimum quality standards and indicators for community engagement. UNICEF, New York

[23] Van der Roest HG, Prins M, van der Velden C, Steinmetz S, Stolte E, van Tilburg TG, de Vries DH (2020) The impact of COVID-19 measures on well-being of older long-term care facility residents in the Netherlands. J Am Med Dir Assoc 21(11):1569–1570 33036911 10.1016/j.jamda.2020.09.007 PMC7833500

